# Plant age at the time of ozone exposure affects flowering patterns, biotic interactions and reproduction of wild mustard

**DOI:** 10.1038/s41598-021-02878-9

**Published:** 2021-12-06

**Authors:** Laura Duque, Erik H. Poelman, Ingolf Steffan-Dewenter

**Affiliations:** 1 Department of Animal Ecology and Tropical Biology, Biocenter, University of Würzburg, Würzburg, Germany; 2 Laboratory of Entomology, Wageningen University, Wageningen, The Netherlands

**Keywords:** Environmental impact, Plant ecology, Abiotic

## Abstract

Exposure of plants to environmental stressors can modify their metabolism, interactions with other organisms and reproductive success. Tropospheric ozone is a source of plant stress. We investigated how an acute exposure to ozone at different times of plant development affects reproductive performance, as well as the flowering patterns and the interactions with pollinators and herbivores, of wild mustard plants. The number of open flowers was higher on plants exposed to ozone at earlier ages than on the respective controls, while plants exposed at later ages showed a tendency for decreased number of open flowers. The changes in the number of flowers provided a good explanation for the ozone-induced effects on reproductive performance and on pollinator visitation. Ozone exposure at earlier ages also led to either earlier or extended flowering periods. Moreover, ozone tended to increase herbivore abundance, with responses depending on herbivore taxa and the plant age at the time of ozone exposure. These results suggest that the effects of ozone exposure depend on the developmental stage of the plant, affecting the flowering patterns in different directions, with consequences for pollination and reproduction of annual crops and wild species.

## Introduction

Air pollution is an anthropogenic driver affecting the health of both humans and terrestrial ecosystems^1–3^. Tropospheric ozone is a highly oxidative atmospheric pollutant that has the potential to change a plant’s metabolism and therefore its interactions with other organisms^1^.

The formation and removal of tropospheric ozone is complex and it is in a constant balance that depends on pollutant emissions as well as climate and meteorology^4^. Tropospheric ozone is a secondary pollutant, formed by the reaction of primary pollutants, namely nitrogen oxides (NOx) and volatile organic compounds in the presence of sunlight. It can be removed by NO (nitric oxide) titration, photolysis, deposition on surfaces and uptake by plants^4^. Tropospheric ozone concentrations have greatly increased since pre-industrial times^5^. While the effort to reduce precursor emissions has led to a reduction of tropospheric ozone levels in some parts of the world^6,7^, globally, ozone concentrations are still increasing^8^, and in places like China, it is now an issue of major concern^9^. Due to the highly complex balance of production and removal, ozone concentrations can increase even when air pollution in general decreases, as was the case during the first COVID-19 lockdown in some cities in Europe and China^10^ and can be higher away from the places where the majority of the primary pollutants are emitted, like rural areas^4,7^, where it can affect vegetation.

In an ozone-polluted atmosphere, a plant’s susceptibility and reaction to ozone may determine its competitiveness and therefore its persistence as part of a given plant community^11–13^. Ozone pollution also alters root exudation, modifying soil microbial communities^14,15^; it alters the nutritional value of plants, affecting the development of herbivores feeding on ozone exposed plant tissues^16–19^; and it changes plant volatiles, potentially altering plant attractiveness or repellence to insects^20–24^.

Reproductive performance depends not only on the plant and its capability to react to a multitude of abiotic environmental factors^25–27^, but also on the interactions of the plant with other organisms in its ecosystem^28^. Tropospheric ozone can reduce plant reproductive performance, as measured by the number of seeds produced by the plant, in a wide range of species^26,29^. Some of the proposed mechanisms by which ozone might affect reproductive performance include (1) decreased photosynthesis by means of (a) reduced photosynthetically active leaf area due to cell death or accelerated senescence or (b) decreased Rubisco activity, (2) inhibition of assimilate translocation and (3) effects on reproductive processes such as decreased pollen germination, decreased pollen tube growth and abscission of reproductive sites^26,30^. However, other factors should be taken into consideration, such as plant phenology, as well as interactions of plants with other organisms, namely their pollinators and herbivores. Ozone has been shown to alter the timing of the onset of flowering^31–33^ and to decrease the number of flowers^32,33^, which may alter reproductive success. For entomophilous plants (i.e. plants that depend mainly on insects for pollination) interaction with pollinators is key to their reproductive performance^34,35^. Ozone can affect the floral volatiles that the plant emits to attract pollinators^24^, or posteriorly react with those volatiles in the atmosphere^36^. These changes in floral volatiles render the floral scent less attractive for naïve pollinators, but the pollinators may have the ability to learn these new scents^37^. However, few studies have addressed the subject of the impact of ozone on the interaction between plants and their pollinators from an empirical point of view. Another group of organisms that may strongly affect a plant’s performance are herbivores, either by feeding on the plant vegetative parts and reducing plant photosynthesis, or by directly destroying reproductive organs or fruits. Most studies that assessed the effects of tropospheric ozone on plant-herbivore interactions have focused their attention on the effects of the modified interactions from the perspective of the herbivores ^16–19,38^, while the consequences for plant reproduction are little explored. Since Lee et al.^39^ suggested that sensitivity of plants to ozone depended on the timing of exposure, a handful of studies assessed the effects of plant age/phenological stage during exposure on reproduction and other plant parameters^40–43^ and this factor was included in some ozone-exposure indexes^44^. However, no single study tried to assess the effects of the timing of exposure to ozone on plant-insect interactions and the consequences for the reproductive performance of the plants.

In the present study, we exposed wild mustard (*Sinapis arvensis* L.) plants, in fumigation chambers, to an acute ozone exposure in four different periods of their life cycle. These plants were subsequently grown in a field where they were accessible for both pollinators and herbivores. We aimed to answer the following research questions: (1) Does an exposure to enhanced levels of ozone alter the reproductive performance of wild mustard plants and does this depend on the plant age at the time of exposure? (2) When reproductive performance is affected, is this due to compensatory responses in the plant’s phenology? (3) Is this due to altered interactions between the plant and its pollinators? (4) Is this due to altered interactions between the plant and its herbivores?

## Material and methods

**Figure 1:**
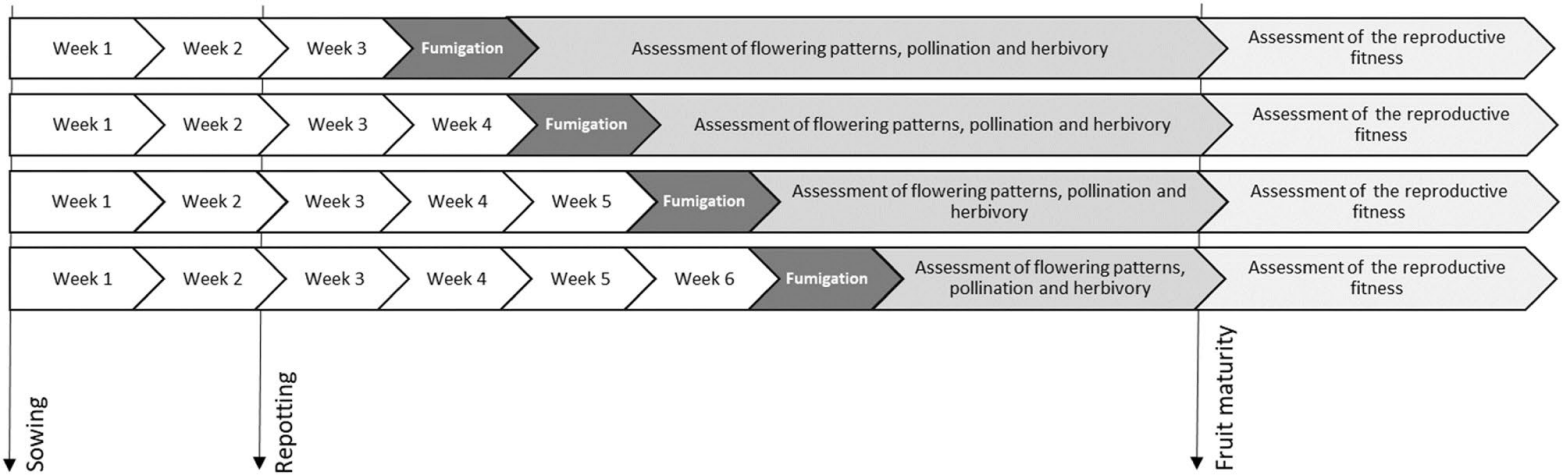
Chronology of the experiment.

This experiment was performed between April and October 2017. Wild mustard (*Sinapis arvensis* L.) plants were exposed to a target level of 120 ppb of ozone in a fumigation system placed in a greenhouse. Plants were exposed at different ages (Fig. 1). Following exposure, plants were placed outdoors to be openly exposed to pollinators and herbivores. Plant reproductive performance was assessed. All methods were performed in accordance with the relevant guidelines and regulations.

### Plant material

Native to Europe, the Middle East and Western Asia, wild mustard is now widespread through most of the temperate regions of the world, where it is considered a weed^45^. It is an annual entomophilous plant with an indeterminate growth habit (i.e. it continues to produce flowers when the reproductive success is low)^45,46^. As a plant whose germination starts mainly in early spring^47^, wild mustard is at risk of increased ozone exposure, as the timing of maximum exposure to ozone is shifting from Summer to Spring^48^.

For this study, wild mustard seeds were provided by the Botanical Garden of Konstanz, Germany. The seeds were sown in seed trays and repotted 2 weeks later into 18 × 18 cm pots. A 2:1-mixture of peat based substrate (Einheits Erde CL ED 73) and sand (Hamann Filtersand 0,7–1,25 mm) was used both in the seed trays and in the pots. The plants were watered throughout the experiment according to their needs and no fertilizer was added. 3 cohorts of plants were sown on the 26th April, 14th June and 12th July 2017. 32 plants were used per cohort. The 1st cohort was kept in a greenhouse at all times until after fumigation. This cohort of plants was found to be very tender, breaking easily when put outside after fumigation. Therefore, for the 2nd and 3rd cohorts, the plants were often taken outside of the greenhouse before fumigation, during daylight hours, for hardening off.

To test whether the effects of ozone are dependent on plant age at the time of fumigation, plants were fumigated at different ages, with plant age being counted from the day of sowing. Hence, each week since the plants were 3 weeks old up until when they were 6 weeks old, 8 plants (4 per treatment level) were chosen for fumigation. Plant selection and assignment of fumigation treatment were random, except that it was made sure all plants in one round of fumigation were in the same phenological stage. Plants from the first cohort developed more slowly, presumably due to the meteorological conditions observed, therefore plant age and plant phenological stage at the beginning of the fumigation treatment are mostly overlapping but do not totally coincide for all cohorts (Table S1).

### Ozone fumigation

Fumigation took place in a fumigation system with 2 glass chambers placed in a greenhouse^18^. In this fumigation system, the air is supplied by a compressed air system and passes through an activated charcoal filter and a particle filter before it reaches the chambers. To increase the ozone level, a small portion of the incoming air diverges from this main stream and passes through an air dryer (AIRdyer3.1, INNOTEC) and a customized ozone generator (INNOTEC high engineering GmbH) before it reaches one of the chambers. To attain the desired ozone-enhancement, the ozone generator is regulated by a controller connected to an ozone analyser (APOA-370, Horiba Ltd). The air comes into the chambers through an opening at the top of the door frames and leaves the chambers passively through an opening at the bottom of the door frames. The air that is analyzed derives from two different points at different heights in the chambers. When the air is not being ozone-enhanced, the ozone concentration is below 1 ppb.

The fumigation treatment consisted of exposing plants to 2 different levels of ozone: ozone-clean and ozone-enhanced. The chambers were randomly assigned an ozone level before each fumigation round. The ozone-enhanced level (henceforward called ozone) consisted of exposing the plants to a target ozone concentration of 120 ppb of ozone for 6 h/day from 11:00 to 17:00 CEST and to < 1 ppb of ozone for the rest of the day. Due to some technical problems, the ozone concentration during the fumigation was very variable, particularly for the first plant cohort (Table S1). In the ozone-clean level (henceforward called control) the plants were exposed to < 1 ppb of ozone all day long. The fumigation treatments lasted 7 consecutive days.

120 ppb ozone is a very high tropospheric ozone concentration, but one that is sometimes exceeded during high ozone episodes in the current times and is expected to occur more often in the future in the developing regions of the world^49^. However, these exact conditions are unlikely to occur, as in nature, ozone concentration will not rise from < 1 ppb to ~ 120 ppb in a matter of minutes or drop again to < 1 ppb in a short period. On the other hand, the control treatment does not represent the ambient ozone levels currently observed in the troposphere, but the absence of ozone.

### Pollination and herbivory

At the end of each fumigation, the plants were placed outside in an experimental garden. The plants were kept in the pots and the pots were ~ 80% buried in the ground. The plants from each cohort were randomly positioned in an 8-row rectangular design with 2 m distance between plants.

During flowering, plants were observed for 4-min periods, ~ 3 times per week, for flower visits. We did 19 ± 5 observations per plant for a total of 1588 4-min observations. The sequence of plants observed on each day was randomized. During these observations, we recorded the number of flower visitors, the visitor guild (wild bee, honeybee, bumblebee, syrphid fly, other flies, butterflies and others), and, when possible, the number of flowers visited. Ants and rape pollen beetles were also found on the flowers, but they were disregarded, for our purpose was to assess possible pollinator visitation and they are not expected to provide pollination services. In more than 90% of the observation periods, the number of open flowers per plant was also counted. Missing data on the number of open flowers was predicted by fitting a flowering curve for each plant using the gam function (mgcv package version 1.8–31^50^) in R .

The plants were observed 1–2 times per week for the presence of herbivores and predators (abundance) or signs of herbivory (percentage of leaf tissue damaged by chewing herbivores). When abundance of herbivores was above circa 200 (this was the case for aphids only), abundance was estimated by considering the area occupied by a subset of herbivores. Percentage of leaf tissue damaged by chewing herbivores, henceforward referred to as herbivore damage, did not include leaf mining.

### Reproductive success

At fruit maturity, the number of seeds produced per pod was counted for a set of 20 pods per plant (when possible) and 100 seeds per plant (when possible) were weighed. The plants were collected and the total number of fruits produced per plant, as well as the total number of reproductive sites that did not set fruit, were counted. Using the data collected, we estimated the total number of seeds (= number of fruits produced × average number of seeds per pod) and the total seed weight (= estimated total number of seeds × average weight of one seed) produced by the plant. The 10 plants that produced an estimated number of seeds lower than 100 were not considered in the statistical analysis of this study, because we considered they had an abnormal development (number of seeds per plant was 4778 ± 2477 (mean ± sd)).

### Statistical analysis

All statistical analyses were performed in R version 4.0.1^51^. To answer our research questions, we fitted (generalized) mixed effects models using the glmmTMB function from the glmmTMB package version 1.0.1^52^. The fit of the models was tested using the Dharma package version 0.3.1^53^. For all models, we used the factors treatment (control vs ozone), plant age at the time of exposure and their interactions as fixed factors and plant cohort as a random factor. Other variables were only included when they improved the fit of the models (see Table S2 for further details on the models). Furthermore, we used AICc (Akaike Information Criterion, corrected for small sample sizes) to select the best model predicting the number of seeds produced per plant. Post-hoc tests were performed to assess at what plant age exposure to ozone is relevant. Post-hoc tests as well as model predictions presented in the graphs, were obtained using the emmeans and emtrends functions in the emmeans package version 1.4.7^54^.

## Results

**Figure 2:**
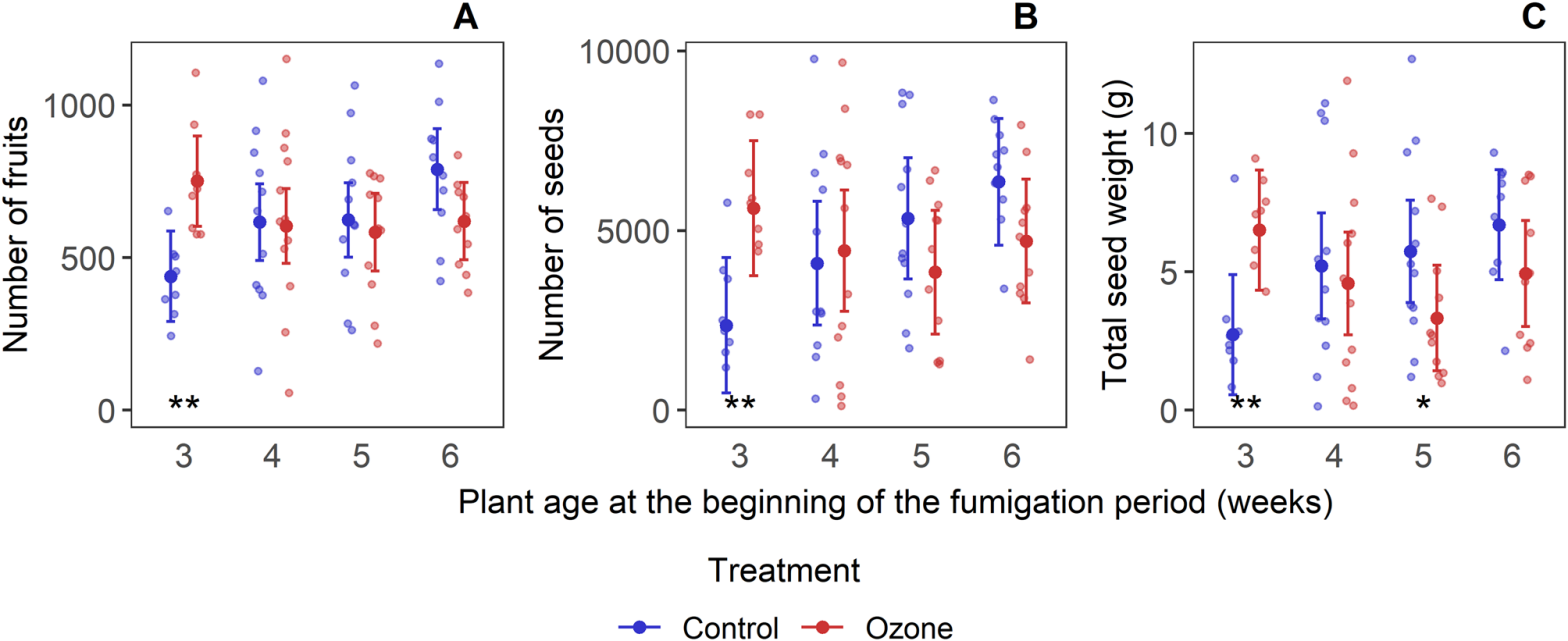
The effect of exposure to ozone at different plant ages on the reproductive performance of individual plants. The dots are data points and the error bars represent the means and standard deviations as provided by the models tested. The asterisks represent significant effects of ozone exposure within plant age (*0.05 ≥ *P* ≥ 0.01; **0.01 > *P* ≥ 0.001).

Wild mustard plants were affected by ozone differently in their reproductive performance depending on the age of the plant at the beginning of the fumigation period (Treatment x Plant age: *P* = 0.006, *P* < 0.001 and *P*  = 0.003, for the number of fruits, number of seeds and total seed weight produced, respectively, Fig. 2, Table S3). Reproductive performance of control plants differed between plants exposed at different ages likely due to the different amounts of time they were kept in the more favorable greenhouse conditions. In 3-week old plants, ozone exposure improved the reproductive performance of plants: they produced 1.7 times more fruits, 2.4 times more seeds and 2.4 times higher total seed weight than the respective control plants (*P* = 0.004, 0.002 and 0.006, respectively, Table S3). The plants showed a tendency to have reduced reproductive performance when fumigated at later ages, particularly when fumigated at age 5 and 6 weeks. This tendency was only significant for the total seed weight of plants exposed at 5 weeks, when the yield of ozone-exposed plants was only 58% of the control plants (*P* = 0.035). The results from flowering phenology, plant-pollinator and plant-herbivore interactions provide possible explanations for these plant age-dependent responses to ozone stress.

### Flowering phenology

**Figure 3:**
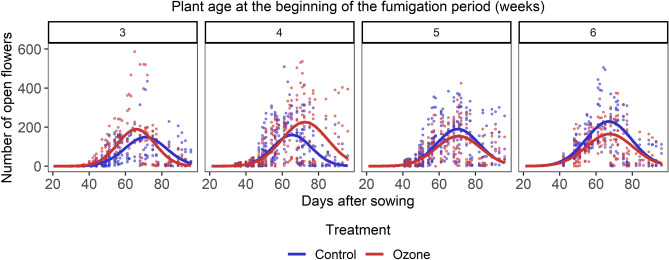
The effect of exposure to ozone at different plant ages on the flowering patterns of individual plants. The dots are data points and the curves are the regression lines as provided by the model.

Ozone exposure affected the flowering patterns of the plants differently according to plant age (Treatment x Plant age, *P* = 0.027; Treatment x Plant age x DAS (Days after sowing), *P*  <  0.001, Fig. 3, Table S4). Plants that were exposed to ozone when they were 3 weeks old had, on average, more open flowers on the observation days than control plants (*P* = 0.008, Table S5). In addition, exposure to ozone modified the flowering curve of plants exposed when they were 3 and 4 weeks old (*P* < 0.001, Fig. 3, Table S5). Ozone exposure lead to earlier flowering of plants exposed at 3 weeks, while plants exposed when they were 4 weeks old started flowering at the same time as control plants but reached peak flowering later and prolonged the flowering period (Fig. 3). There was no significant effect of ozone on the flowering patterns of plants fumigated at later ages, but we observed a tendency for a decrease in the number of open flowers (*P* = 0.285 and *P*  = 0.062 for plants fumigated at ages 5 and 6 weeks respectively, Table S5, Fig. 3).

### Plant-pollinator interactions

Of the registered 3150 flower visitors, 90% were bees and syrphids (69 and 20%, respectively). The other flower visitors were flies (7%), butterflies (1%) and other insects (2%). Amongst bees, 66% were honeybees and 34% were wild bees, while the representation of bumblebees was lower than 1%. The response of the flower visitors to the ozone treatment depended on the plant age at the beginning of the fumigation period (Treatment x Plant age: p < 0.001, Table S6). In general, there was a tendency for plants that were fumigated with ozone at earlier ages to have more flower visitors than the respective controls, while this tendency was reverted for plants fumigated at later stages (Table S6 and Fig. S1). However, the response varies with the pollinator guild considered, with bees showing this pattern more clearly than syrphids or the overall visitors (Table S6 and Fig. S1). When refitting the models tested by correcting for the number of open flowers, no effects of ozone on the number of visitors were observed, except for the group of large syrphids, that still showed a positive effect of ozone exposure at the 3 weeks stage on flower visitation (p = 0.021, Table S7). This indicates that the effects of ozone on the number of flower visitors were mainly due to the changes in the number of open flowers. The number of flowers visited by a single visitor in a 4-min interval was not affected by ozone (Treatment: p = 0.726), independently of the timing of exposure to ozone (Treatment x Plant age: *P* = 0.462).

### Plant-herbivore interactions

Several herbivores and predators were observed on the plants after they were placed in the field. Although we often observed butterfly eggs of the species *Pieris rapae* and *Pieris brassicae*, we rarely saw any caterpillars, indicating high predation rates or plant resistance to insect eggs. The most abundant herbivores were aphids, especially of the species *Brevicorine brassicae* and *Myzus persicae*. Other insects that we observed in greater numbers on the plants included *Lygus pratensis* bugs and larvae of the aphid midget *Aphidoletes aphidimyza.* Although ozone did not have a strong effect on the level of damage by chewing herbivores (Treatment: *P* = 0.127, Treatment x Plant age: *P* = 0.263, Table S8), the damage tended to be higher on plants fumigated with ozone at plant age 6 weeks than on control plants (*P* = 0.015, Table S8, Fig. S2). Also, on the rare occasions when ozone had an effect on insect abundance, the effect was positive for herbivores and negative for predators (Table S8, Fig. S2). The number of aphids was higher on plants fumigated with ozone at plant age 4 weeks (*P* = 0.041), the number of *Lygus pratensis* bugs was higher on plants fumigated with ozone at plant age 3 weeks (*P* = 0.049), but the number of aphid midget larva was lower on plants fumigated with ozone at plant age 6 weeks (*P* = 0.015), than on the respective controls.

**Figure 4:**
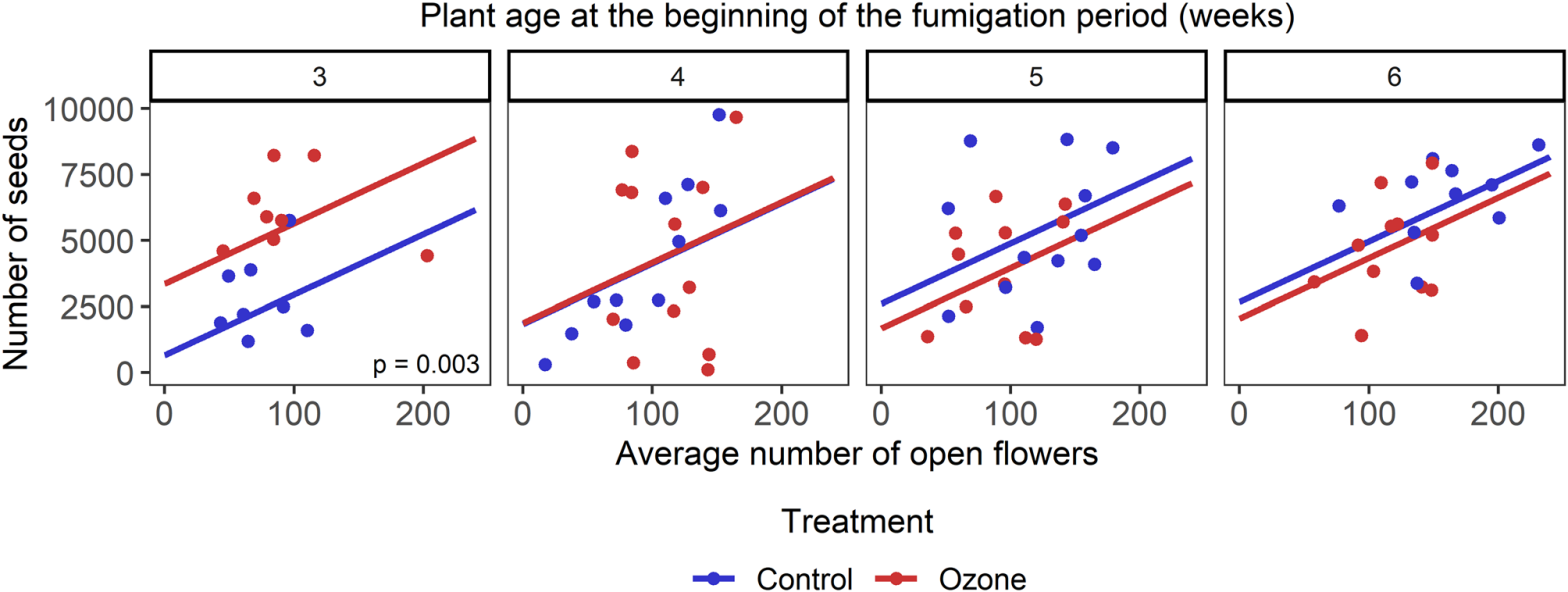
The effect of ozone exposure at different plant ages on the number of seeds produced by the plants in relation to the average number of open flowers recorded during observations days. The lines represent the best model tested (lowest AICc) for predicting the number of seeds. Regression lines are provided by the emmeans package. *P*-values are presented for the effect of ozone when *P* ≤ 0.05.

Including herbivory (average number of aphids and average herbivore damage), pollination (average visitation rate) and average number of open flowers as predictor variables in the model predicting the number of seeds produced by the plants improved the model fit, while adding only the average number of open flowers provided the best model (Table S9). The number of seeds produced by the plant increased with increased number of open flowers on the plants (*P* < 0.001, Table S9, Fig. 4). Including the average number of open flowers in the model resulted in a weaker pattern of the direct effects of ozone on the number of seeds produced (Treatment x Plant age: *P* = 0.018, Table S9), while still showing a strong positive effect of ozone exposure on plants fumigated when they were 3 weeks old (*P* = 0.003, Table S9, Fig. 4). This indicates positive effects of ozone on the number of seeds produced that go beyond the increase in the number of flowers.

## Discussion

Subjecting wild mustard plants to an acute ozone exposure at different stages of their life revealed that ozone had opposite effects on plant reproductive performance depending on plant age at the time of exposure. While younger plants tended to overcompensate, meaning they responded to ozone stress by increasing reproductive performance, older plants were less plastic in their responses and tended to show reduced reproductive performance. By analyzing possible underlying mechanisms that led to these differences, we could reveal that 1) the effects of ozone on the flowering patterns of the plants depended on plant age during exposure, 2) the number of pollinators visiting the plants depended on plant age during exposure and was mostly driven by the different number of flowers open for pollination, 3) ozone stress tended to increase herbivory, particularly the abundance of sucking herbivores, but variation among taxa was large, 4) the higher reproductive performance of plants exposed in early ages was mostly driven by increased production of flowers.

Plant reproduction was affected by ozone, but the direction of the effects depended on the age of the plants at the time of exposure. Number of fruits, number of seeds and total seed weight produced by the plants was higher on plants exposed to ozone when they were 3 weeks old than on the respective controls, while exposing plants to ozone later in their life cycle tended to have the opposite effect. Studies reporting positive effects of acute ozone exposure on plant reproduction are uncommon^26^. Previous studies that assessed the influence of plant age/developmental stage on the effects of ozone on *Plantago major* showed only negative effects of ozone on reproduction and only when exposure occurred during the early stages of flowering^40,41^. Here, we observed a reduction in total seed weight of plants that were exposed to ozone when they were 5 weeks old. These plants were in the inflorescence emergence stage at the beginning of fumigation, but before the end of the fumigation period most of the plants had started flowering. This shows that plant reproductive sensitivity to ozone is the highest in the beginning of flowering also in wild mustard. However, it is possible that the age-dependent effects of ozone on plant reproduction are not only related to plant phenological stage during exposure but also to the changing conditions in the canopy as plants grow. We registered an increase in mean air relative humidity in the fumigation chambers as plants were fumigated at increasingly older ages (Table S1). This is likely a result of increased transpiration due to increased leaf area index^55^, which in turn is related to plant growth/age. In conditions of increased relative humidity, plants tend to open the stomata, increasing stomatal conductance and therefore ozone uptake^56^. Increased ozone uptake is associated with stronger negative effects of ozone on vegetation^57^. Thus, ozone uptake would be higher when plants are fumigated at older ages, which would explain the tendency for negative effects of ozone on the reproduction of plants fumigated at older ages.

We further investigated what could be at the origin of the observed differences in the response of plants exposed to ozone at different plant ages on reproduction. Analyzing the flowering patterns of the plants, we observed that plants exposed to ozone when they were 3 weeks old had more flowers than the respective controls, with a tendency for the direction of these effects to be reverted as the plants were exposed at progressively older ages. Previous studies that assessed the number of flowers of plants exposed to ozone showed that this pollutant either did not change or reduced the number of flowers^32,33^. In the light of our results, this could be due to the fact that these observations were made on older plants. However, the ozone fumigation in the referred studies corresponded to a long-term exposure of the plants and the plants used were perennials, which do not necessarily show the same sensitivity to ozone as annuals like wild mustard^11,26^. Also, having an indeterminate growth habit, wild mustard may have higher chances of compensating for negative effects of abiotic stresses than determinate plant species^58^, although reproductive growth habit alone does not explain the species-specific effects of ozone on the number of flowers^26^. In our study, 3-week-old exposed plants started flowering earlier when fumigated with ozone, while 4-week-old exposed plants showed prolonged flowering. In a previous study we had already observed that ozone promoted earlier onset of flowering in wild mustard^31^. Although in that study the fumigation was performed when the plants were 4 weeks old, on average they were likely in an earlier stage in their life cycle than in the present study due to the observed conditions during their development. Earlier flowering onset is a common phenomenon when plants are under abiotic stresses, such as poor nutrition, drought, high salinity and high or low temperatures^59^, but it can also be observed on plants exposed to biotic stresses, such as herbivory and damage caused by pollinators^60,61^. Together, our results suggest that compensatory stress responses of young wild mustard plants in the ozone treatment lead to higher investment in flower production, together with earlier flowering or extended flowering times. In contrast, stress responses of more mature annual plants and of perennial plants are less plastic and cannot compensate for negative effects of ozone stress.

In our experiment, we were also interested in assessing other indirect effects of ozone stress on plant reproductive performance, namely those related to mutualistic and antagonistic plant-insect interactions. The ozone fumigation and the timing of the exposure affected the number of flower visitors on the plants. Although the results depended on the pollinator guild considered, there was an overall tendency for positive effects of ozone on the number of visitors received by plants exposed at younger ages (3- and 4-week-old), but negative effects on plants exposed at older ages (5- and 6-week-old). When correcting for the number of open flowers, these effects were no longer detectable, suggesting that the changes in the number of flower visitors was driven, in this experiment, by the change in the number of open flowers^31^. In the present study, an exception was observed for the number of large syrphid flies visiting the plants, which was still higher on plants exposed to ozone when they were 3 weeks old than on the respective controls after the correction for the number of open flowers. These large syrphid flies possibly used other floral traits besides flower number as a cue for the quantity or quality of the rewards available in the plants. Stabler^62^ showed that exposure of broad bean plants to ozone reduced the amount of pollen produced and decreased its protein content, while the production of floral nectar was increased by short-term exposures and the nectar had a higher concentration of sucrose and amino acids when the exposure was interrupted at flowering. Pollinator requirements are species-specific. Unlike bees, that need to provide their offspring with enough resources to sustain them through the larval stage and, therefore, need to forage for higher quantities of protein in the form of pollen, syrphid flies, and especially large syrphid flies, consume pollen mainly for the maturation of the reproductive organs, therefore foraging mostly for nectar, their source of energy to fuel flight and increase longevity^63^. Therefore, we consider that the possible effects of ozone on the quantity and quality of nectar may have served as attractants to this group of foragers, possibly by encouraging revisits of previously visited plants. While ozone was shown to change the profile of floral scents emitted by the plants, this effect seems to be absent in wild mustard, that maintains both its floral volatile blend composition and rate of emission^24^, which might explain the absence of stronger plant-mediated effects of ozone on flower visitation.

The plant-mediated effects of ozone on herbivory were less clear than those on flower visitation, but in the few situations where effects were observed, they were positive, meaning ozone increased herbivory in exposed plants. This seems to contradict most studies on effects of ozone on the oviposition preference of herbivores, which report a reduced preference for ozone-exposed plants^18,64–66^. However, several studies refer to a reduction in the nutritional quality of leaves of plants exposed to elevated levels of ozone^16,17,19^, which may lead to compensatory feeding mechanisms, while other studies suggest an increase in leaf palatability^67^. In both cases there may be an increase in consumption of leaf material^19,65,67–69^, therefore increasing the damage by chewing herbivores, a general tendency observed in this study that was only significant for plants exposed to ozone at age 6-weeks. The plant-mediated effects of ozone on the preference and performance of aphids are more ambiguous and depend on the level of exposure and the age of the exposed plants^70^. The latter was the case in our study, with higher numbers of aphids on plants exposed when they were 4 weeks old than the respective controls, but no difference being observed when plants were exposed to ozone at other ages. Holopainen and Kössi^71^ observed that ozone can stimulate aphid population growth of aphids feeding on spruce seedlings exposed to 80 ppb of ozone, but aphids are not affected when the host plants are exposed to other levels of ozone or when the exposure continues until shoot maturity. Other studies showed that aphids tolerate well the plant-mediated effects of ozone and are not affected by the exposure^72–74^. We also observed that the number of *Lygus pratensis* bugs was higher on plants exposed to ozone when they were 3 weeks old. This contrasts with the results obtained by Manninen et al.^72^ for another *Lygus* species (*Lygus rugulipennis*), whose performance was reduced in ozone-exposed Scots pine seedlings. Except for the study of Telesnicki et al.^74^ that used *Triticum aestivum* as the plant species, all other studies mentioned above on sucking herbivores were performed with perennial plant species, meaning that the longevity of the plant may influence its ozone plant-mediated effects on the performance of the herbivores.

On the other hand, predation, as measured in this study by the number of aphid midget larvae, followed the opposite trend of herbivory, with lower numbers of these larvae being observed on plants exposed to ozone at plant age 6-weeks than on the respective controls. Vuorinen et al.^75^ did not observe any changes in attraction of predatory mites following plant exposure to ozone, since ozone-exposed plants emitted volatiles were similar to those of plants infested with spider mites. Studies on the effects of ozone on multitrophic interactions focused mostly on parasitism of insect herbivores and the potential of ozone to disrupt the attraction of parasitoids due to chemical reactions with signaling volatiles in the atmosphere^76,77^, or the potential of ozone to lead to plant volatiles emissions that are similar to those of herbivore-infested plants^64^. Studies on the plant-mediated effects of ozone on insect herbivore predation are, however, largely missing and require further investment.

Including flowering, pollination and herbivory as potential co-variables in the model predicting the number of seeds produced per plant demonstrated that the number of flowers per plant is a key factor that shows indirect effects of ozone. Furthermore, the increase in the number of open flowers that results from exposing the plants to ozone at early ages is one way the plant reallocates its resources to reproduction.

In nature, it is possible that plants will experience high ozone levels at several points during their life cycle. Future studies should contemplate this hypothesis and try to assess whether the flower-stimulating effects observed in plants exposed at early ages would be counterbalanced when the plants would be exposed to a second ozone episode later in their lives. Also, studies in a more natural setting and studies including a wider variety of plant exposures to ozone (acute vs chronic) should be performed in order to assess if comparable results are observed.

In this study, we used a stream of compressed air as the feed gas for the ozone generator. Silent discharge ozone generators produce small amounts of nitrogen oxides when using dry air as the feed gas, particularly N_2_O_5_ (dinitrogen pentoxide)^78^. It could be argued that this way of generating ozone produces confounding effects, making it hard to disentangle whether the effects observed are related to the increase in ozone concentration or to unwanted by-products of the ozone generation. However, the increase in concentration of these by-products is kept two orders of magnitude below the ozone concentration^78^. Furthermore, there is little evidence that the amount of N_2_O_5_ produced would have strong effects on plants’ development and growth, although some effects on the amounts of leachable nitrate and some other ions have been observed in Norway spruce needles when using ozone concentrations 2.5 times higher than the one used in our study^79^. However, to avoid the uncertainty of whether ozone generation by-products are interfering in the results, future studies should use pure oxygen as the feed gas in silent discharge ozone generators.

Overall, our study shows that, along with ozone exposure levels, conditions during exposure and susceptibility of plants, the plant age/ plant phenological stage at the time of exposure is also key to understanding the effects of ozone on reproductive performance. Plant age does not only affect the susceptibility of the plants but also the direction of the effects of ozone. In this study, an acute exposure to ozone at an earlier age resulted in higher reproductive performance of wild mustard plants while plants exposed later showed a tendency for the opposite effect. Also, the changes in the number of flowers provided a good explanation for both the changes in reproductive performance and the changes in pollinator visitation.

## Supplementary Information

Supplementary Information.

## Data Availability

The data generated during the current study are available from the authors on reasonable request.
