# Supplementary material for: Plant age at the time of ozone exposure affects flowering patterns, biotic interactions and reproduction of wild mustard
Source: Sci Rep. 2021 Dec 6;11:23448. doi: 10.1038/s41598-021-02878-9 (PMC8648743; doi:10.1038/s41598-021-02878-9)
Supplement: Supplementary file 1 — Supplementary Information. [file 41598_2021_2878_MOESM1_ESM.pdf]

Plant age at the time of ozone exposure affects flowering patterns, biotic interactions and reproduction of wild mustard (Supplementary Information)

Laura Duque[1*], Erik H. Poelman[2], Ingolf Steffan-Dewenter[1]

[1] Department of Animal Ecology and Tropical Biology, Biocenter, University of Würzburg, Würzburg, Germany

[2] Laboratory of Entomology, Wageningen University, Wageningen, The Netherlands

***** Correspondence and requests for materials should be addressed to Laura Duque (laura.duque@uni-wuerzburg.de)

Table S1 – Plant phenological stage at the beginning of each fumigation round and conditions in the ozone chamber between 11h00 and 17h00, during the treatment days, for each fumigation round: average ozone concentration, accumulated exposure to ozone (AOT40), air temperature and relative humidity.

Plant cohort	Plant age	Plant phenological stage	[O_3] in the ozone chamber (ppb)	AOT40 (ppb.h)	Air temperature (°C)	Air relative humidity (%)
1	3	Vegetative	95.5 ± 45.0	1872	28.6±1.9	46.0±2.4
	4	Vegetative	97.5 ± 40.5	1530	30.6±2.4	53.4±1.6
	5	Inflorescence emergence	68.9 ± 56.1	791	29.8±1.9	55.6±2.9
	6	Flowering	89.7 ± 44.9	1361	28.0±1.7	69.0±2.9
2	3	Vegetative	113.9 ± 11.9	2277	31.8±2.0	43.7±2.0
	4	Inflorescence emergence	112.2 ± 21.2	2227	29.7±2.5	49.0±2.2
	5	Inflorescence emergence	117.1 ± 10.0	2309	29.6±3.0	57.2±4.3
	6	Flowering	116.7 ± 9.5	2298	29.4±2.5	58.5±4.3
3	3	Vegetative	116.3 ± 11.4	2261	31.4±2.3	41.4±1.5
	4	Inflorescence emergence	117.1 ± 7.0	2310	28.7±2.1	48.6±1.7
	5	Inflorescence emergence	113.9 ± 7.9	2229	28.7±2.3	62.1±2.8
	6	Flowering	116.0 ± 8.7	2271	30.7±2.0	62.2±3.1

Table S2 – Details on the models tested.

Response variable	Predictor variables	Random factors	Overdispersion factors	Zero-inflation factors	Family	Comments
Number of fruits	Treatment<br>Plant age<br>Number of reproductive sites<br>Treatment x Plant age	Plant cohort	----	----	Gaussian	
Estimated number of seeds	Treatment<br>Plant age<br>Treatment x Plant age	Plant cohort	----	----	Gaussian	Mean number of open flowers, mean number of aphids, mean herbivore damage and mean visitation rate were included as possible predictors
Estimated total seed weight	Treatment<br>Plant age<br>Treatment x Plant age	Plant cohort	----	----	Gaussian	
Number of open flowers	Treatment<br>Plant age<br>poly (DAS,2)<br>Treatment x Plant age<br>Treatment x poly (DAS,2)<br>Plant age x poly (DAS,2)<br>Treatment x Plant age x poly(DAS,2)	DAS|Cohort<br>Plant	Treatment<br>Plant age<br>poly (DAS,2)<br>Treatment x Plant age<br>Plant age x poly (DAS,2)	----	Negative binomial 1	
Number of flower visitors	Treatment<br>Plant age<br>Treatment x Plant age	Cohort/DAS<br>Plant	1	1	Negative binomial 1	Models ran with and without offset term (log(Number of open flowers))

Response variable	Predictor variables	Random factors	Overdispersion factors	Zero-inflation factors	Family	Comments
Number of flowers visited	Treatment<br>Plant age<br>Treatment x Plant age	Cohort/DAS<br>Plant	Number of open flowers	----	Negative binomial 2	
Level of chewing-herbivore damage	Treatment<br>Plant age<br>poly(DAS,2)<br>Treatment x Plant age	DAS|Cohort<br>Plant	Plant age<br>poly (DAS,2)	poly(DAS,2)	Gaussian	
Number of aphids	Treatment<br>Plant age<br>poly(DAS,2)<br>Treatment x Plant age<br>Plant age x poly(DAS,2)	DAS|Cohort<br>Plant	Plant age<br>poly(DAS,2)	1	Generalized poisson	
Number of Lygus pratensis	Treatment<br>Plant age<br>DAS<br>Treatment x Plant age	DAS|Cohort<br>Plant	DAS	1	Negative binomial 2	
Number of aphid midget larva	Treatment<br>Plant age<br>poly(DAS,2)<br>Number of aphids<br>Treatment x Plant age	DAS|Cohort<br>Plant	----	1	Poisson	

DAS - Days After Sowing. Poly(DAS,2) indicates that DAS is a quadratic term in the model. Cohort/DAS specifies variable means for each day within each cohort. DAS|Cohort specifies variable slopes for each cohort.

Table S3 – The effect of ozone exposure at different plant ages on the reproductive performance of the plants. Cells shaded in green and red represent positive and negative effects of ozone, respectively.

Response variable	Anova (type II)				Pairwise post-hoc (Control - Ozone)		
	Predictor variable	Chisq	df	p-value	Plant age	t-ratio	p-value
Number of fruits produced	Treatment	0.00	1	0.951	3	-2.97	**0.004**
	Plant age	3.41	3	0.332	4	0.14	0.889
	Number of reproductive sites	3.66	1	0.056	5	0.45	0.652
	Treatment x Plant age	12.47	3	**0.006**	6	1.86	0.067
Estimated number of seeds produced	Treatment	0.06	1	0.811	3	-3.31	**0.002**
	Plant age	6.46	3	0.091	4	-0.42	0.676
	Treatment x Plant age	18.01	3	**<0.001**	5	1.82	0.073
					6	1.90	0.061
Estimated total seed weight	Treatment	0.90	1	0.343	3	-2.82	**0.006**
	Plant age	2.76	3	0.429	4	0.57	0.572
	Treatment x Plant age	14.25	3	**0.003**	5	2.15	**0.035**
					6	1.50	0.138

Table S4 – The effect of ozone exposure at different plant ages on the flowering patterns of the plants. DAS stands for Days After Sowing.

Response variable	Anova (type II)			
	Predictor variable	Chisq	df	p-value
Number of open flowers	Treatment	0.06	1	0.799
	Plant age	19.04	3	**< 0.001**
	poly (DAS,2)	2069.43	2	**< 0.001**
	Treatment x Plant age	9.18	3	**0.027**
	Treatment x poly (DAS,2)	0.16	2	0.921
	Plant age x poly (DAS,2)	55.43	6	**< 0.001**
	Treatment x Plant age x poly(DAS,2)	83.85	6	**< 0.001**

Table S5 – Pairwise comparisons of the flowering patterns of control and ozone-exposed plants within each plant age at the beginning of the fumigation period. The emmeans test analyses how the average number of flowers changes with the exposure to ozone, while the emtrends test takes into account the three-way interaction and compares the slopes of the flowering curves.

Response variable	Pairwise post-hoc (Control - Ozone)		
	Plant age	t-ratio	p-value
	Emmeans		
Number of open flowers	3	-2.64	**0.008**
	4	0.18	0.859
	5	1.07	0.285
	6	1.87	0.062
	Emtrends		
	3	4.53	**< 0.001**
	4	-5.53	**< 0.001**
	5	1.02	0.310
	6	0.03	0.979

Table S6 – The effects of ozone exposure at different plant ages on the number of flower visitors a plant receives in 4-min observation periods. Results from the models that do not consider the offset term for correcting for the number of open flowers. Cells shaded in green and red represent positive and negative effects of ozone, respectively.

Number of visitors in 4-min periods	Not correcting for the number of open flowers						
	Anova (type II)				Pairwise post-hoc (Control - Ozone)		
	Predictor variable	Chisq	df	p-value	Plant age	t-ratio	p-value
All visitors	Treatment	0.03	1	0.868	3	-3.27	**0.001**
	Plant age	5.14	3	0.162	4	-1.64	0.102
	Treatment x Plant age	22.49	3	**<0.001**	5	1.74	0.082
					6	2.45	**0.014**
Bees and syrphids	Treatment	0.01	1	0.909	3	-3.49	**<0.001**
	Plant age	5.25	3	0.154	4	-1.73	0.083
	Treatment x Plant age	25.67	3	**<0.001**	5	2.16	**0.031**
					6	2.39	**0.017**
Only bees	Treatment	0.19	1	0.664	3	-2.96	**0.003**
	Plant age	7.58	3	0.055	4	-2.04	**0.042**
	Treatment x Plant age	27.99	3	**<0.001**	5	2.62	**0.009**
					6	2.88	**0.004**
Honeybees	Treatment	0.09	1	0.766	3	-3.14	**0.002**
	Plant age	11.87	3	**0.008**	4	-1.39	0.166
	Treatment x Plant age	22.39	3	**<0.001**	5	2.65	**0.008**
					6	1.91	0.057
Wild bees	Treatment	0.20	1	0.657	3	-0.91	0.362
	Plant age	2.23	3	0.527	4	-2.22	**0.026**
	Treatment x Plant age	14.65	3	**0.002**	5	1.51	0.132
					6	2.60	**0.009**
Only syrphids	Treatment	0.36	1	0.547	3	-3.12	**0.002**
	Plant age	3.43	3	0.330	4	0.35	0.723
	Treatment x Plant age	10.18	3	**0.017**	5	0.77	0.440
					6	0.26	0.794
Small syrphids	Treatment	0.05	1	0.824	3	-1.66	0.098
	Plant age	3.41	3	0.332	4	1.08	0.282
	Treatment x Plant age	4.65	3	0.199	5	-0.02	0.983
					6	0.89	0.372
Large syrphids	Treatment	0.16	1	0.686	3	-3.42	**<0.001**
	Plant age	1.07	3	0.783	4	0.19	0.846
	Treatment x Plant age	12.93	3	**0.005**	5	1.12	0.265
					6	-0.05	0.958

Table S7 – The effects of ozone exposure at different plant ages on the number of flower visitors a plant receives in 4-min observation periods. Results from the models that correct for the number of open flowers using an offset term. The cell shaded in green represent a positive effect of ozone on the number of large syrphids visiting the flowers.

Number of visitors in 4-min periods	Correcting for the number of open flowers						
	Anova (type II)				Pairwise post-hoc (Control - Ozone)		
	Predictor variable	Chisq	df	p-value	Plant age	t-ratio	p-value
All visitors	Treatment	0.03	1	0.871	3	-0.53	0.594
	Plant age	12.53	3	**0.006**	4	-1.31	0.190
	Treatment x Plant age	4.25	3	0.235	5	0.99	0.321
					6	1.12	0.261
Bees and syrphids	Treatment	0.04	1	0.844	3	-0.90	0.369
	Plant age	11.42	3	**0.010**	4	-1.44	0.150
	Treatment x Plant age	6.40	3	0.094	5	1.52	0.129
					6	1.09	0.274
Only bees	Treatment	0.90	1	0.344	3	-0.31	0.755
	Plant age	9.55	3	**0.023**	4	-1.60	0.111
	Treatment x Plant age	8.82	3	**0.032**	5	1.92	0.055
					6	1.83	0.068
Honeybees	Treatment	0.66	1	0.416	3	-0.96	0.337
	Plant age	7.42	3	0.060	4	-0.94	0.350
	Treatment x Plant age	6.64	3	0.084	5	1.91	0.056
					6	1.34	0.181
Wild bees	Treatment	0.35	1	0.555	3	1.26	0.210
	Plant age	15.48	3	**0.001**	4	-1.72	0.085
	Treatment x Plant age	5.95	3	0.114	5	0.82	0.411
					6	1.06	0.292
Only syrphids	Treatment	1.04	1	0.307	3	-1.57	0.116
	Plant age	7.07	3	0.070	4	0.08	0.935
	Treatment x Plant age	2.45	3	0.484	5	0.12	0.909
					6	-1.00	0.315
Small syrphids	Treatment	0.01	1	0.923	3	-0.53	0.599
	Plant age	8.85	3	**0.031**	4	0.96	0.339
	Treatment x Plant age	2.03	3	0.566	5	-0.87	0.384
					6	0.31	0.761
Large syrphids	Treatment	0.74	1	0.390	3	-2.31	**0.021**
	Plant age	3.64	3	0.303	4	-0.25	0.799
	Treatment x Plant age	6.07	3	0.108	5	0.77	0.444
					6	-0.88	0.380

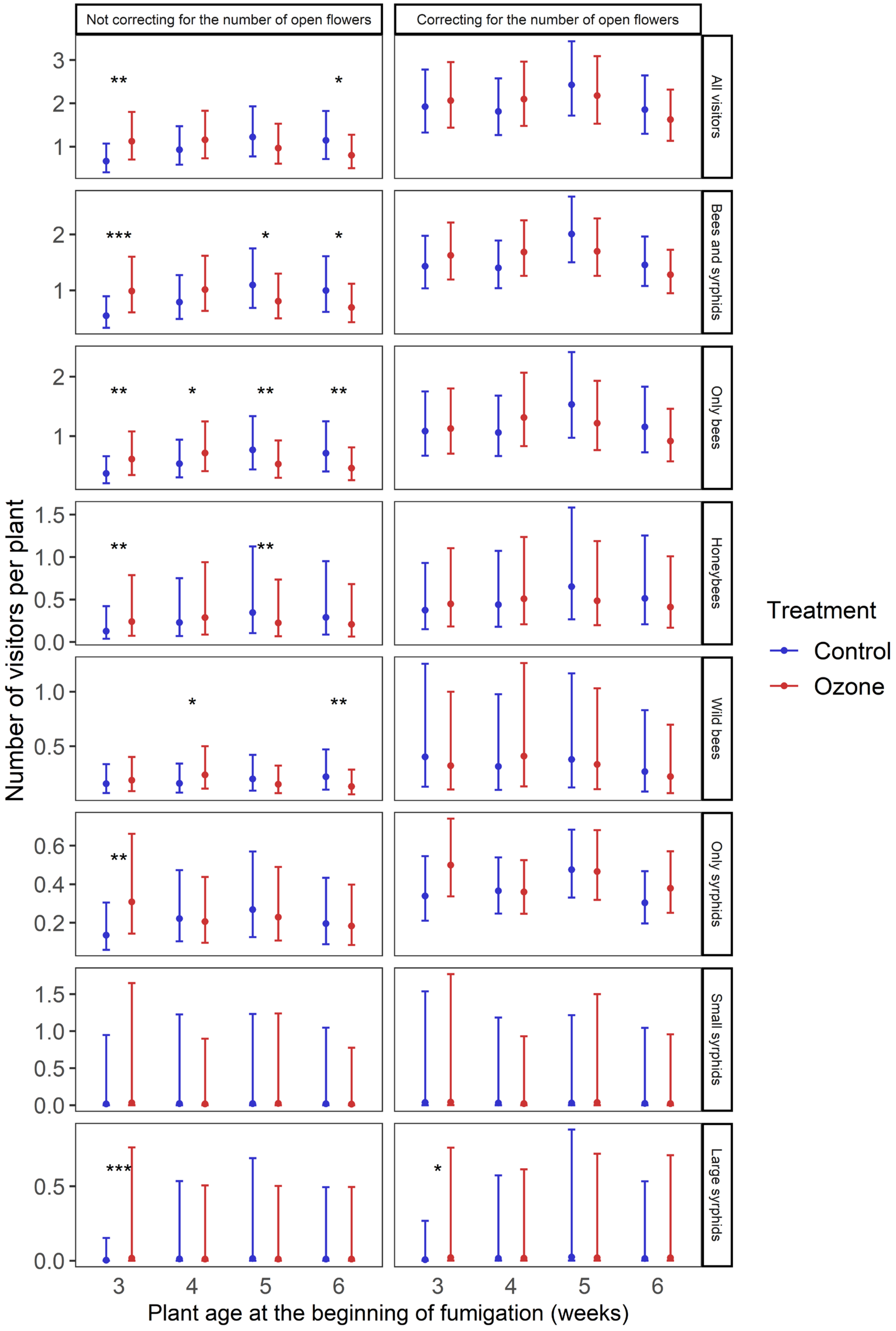

Fig. S1 – The effects of ozone exposure at different plant ages on the number of visitors received per plant in 4-min observation periods. The dots are the means and the bars show the 95 % confidence intervals of the fitted models as provided by the emmeans package. Asterisks represent significant

differences between treatments within a plant age (* - 0.05 > p ≥ 0.01; ** - 0.01 > p ≥ 0.001; *** - p < 0.001).

Table S8 – The effects of ozone exposure at different plant ages on herbivory. Cells shaded in green and red represent positive and negative effects of ozone, respectively. DAS stands for Days After Sowing.

Response variable	Anova (type II)				Pairwise post-hoc (Control - Ozone)		
	Predictor variable	Chisq	df	p-value	Plant age	t-ratio	p-value
Level of damage by chewing-herbivores	Treatment	2.32	1	0.127	3	-0.61	0.544
	Plant age	2.68	3	0.443	4	-0.17	0.869
	poly(DAS,2)	2.23	2	0.327	5	-0.08	0.937
	Treatment x Plant age	3.98	3	0.263	6	-2.43	**0.015**
Number of aphids	Treatment	0.83	1	0.363	3	0.42	0.678
	Plant age	16.78	3	**<0.001**	4	-2.05	**0.041**
	poly(DAS,2)	1.71	2	0.425	5	1.32	0.187
	Treatment x Plant age	7.20	3	0.066	6	-1.40	0.161
	Plant age x poly(DAS,2)	65.59	6	**<0.001**			
Number of *Lygus pratensis*	Treatment	0.09	1	0.766	3	-1.98	**0.049**
	Plant age	2.99	3	0.393	4	1.20	0.232
	DAS	0.40	1	0.525	5	-1.04	0.299
	Treatment x Plant age	7.16	3	0.067	6	0.97	0.335
Number of aphid midget larva	Treatment	0.17	1	0.683	3	-0.59	0.555
	Plant age	2.88	3	0.410	4	-0.16	0.870
	Number of aphids	22.79	1	**<0.001**	5	-0.80	0.424
	poly(DAS,2)	158.06	2	**<0.001**	6	2.44	**0.015**
	Treatment x Plant age	6.81	3	0.078			

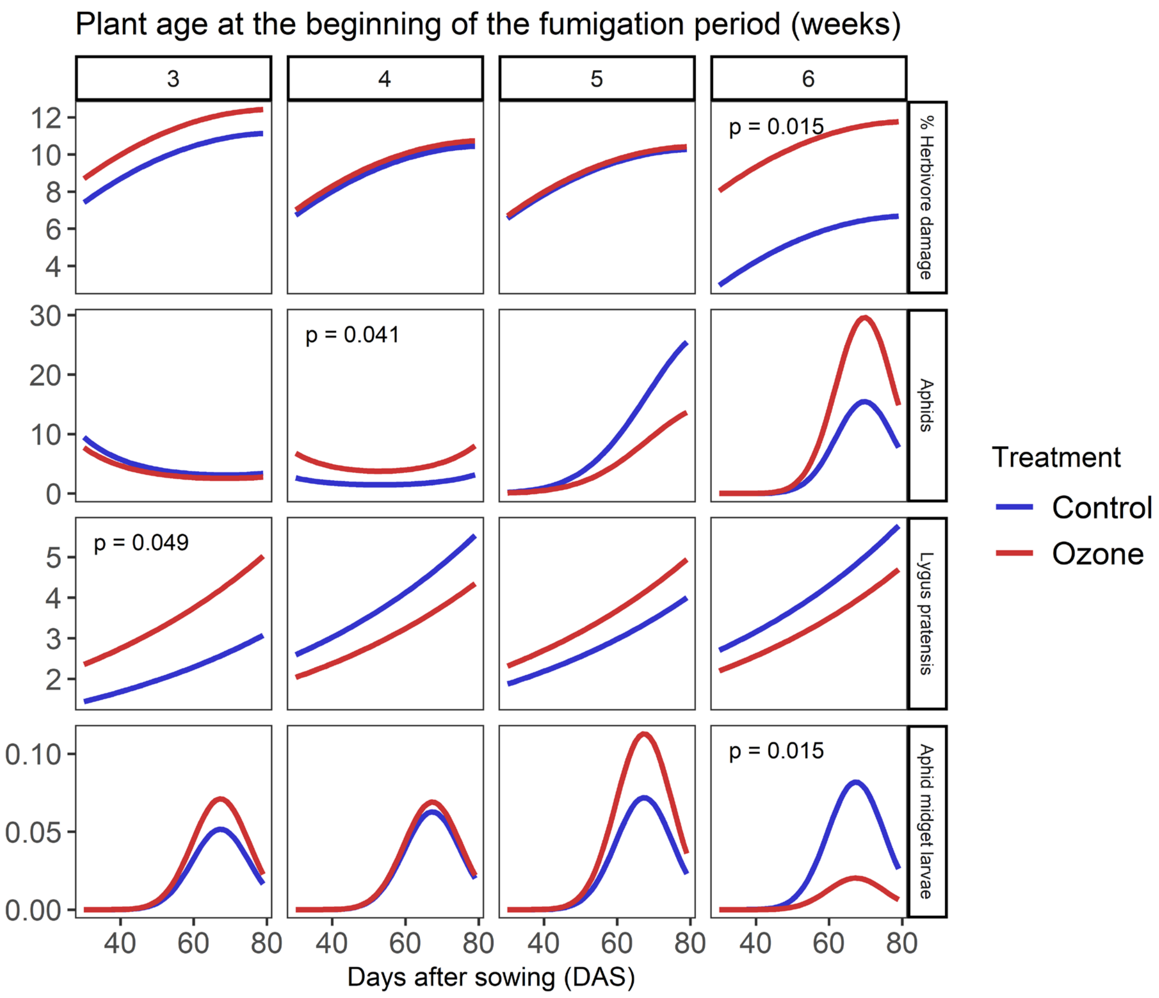

Fig. S2 – The effects of ozone exposure at different plant ages on herbivore damage, and the abundance of two herbivores (aphids and *Lygus pratensis* bugs) and a predator (aphid midget larvae). The lines represent the fitted models as provided by the emmeans package. P-values are presented when the effect of ozone in the post-hoc test was ≤ 0.05.

Table S9 – Results of the models predicting the number of seeds produced per plant, including and excluding herbivory, pollination and flowering parameters as predictor variables. Cells shaded in green and red represent positive and negative effects of ozone, respectively.

Model	ΔAICc	Anova (type II)				Pairwise post-hoc (Control - Ozone)		
		Predictor variable	Chisq	df	p-value	Plant age	t-ratio	p-value
Best model	0.00	Treatment	0.17	1	0.681	3	-2.96	**0.004**
		Plant age	0.42	3	0.936	4	-0.05	0.962
		Average number of open flowers	14.67	1	**<0.001**	5	1.21	0.232
		Treatment x Plant age	10.09	3	**0.018**	6	0.75	0.455
Including all predictors tested	5.55	Treatment	0.04	1	0.852	3	-3.03	**0.003**
		Plant age	0.52	3	0.915	4	0.28	0.784
		Average number of aphids	2.56	1	0.109	5	1.15	0.256
		Average herbivore damage	0.21	1	0.650	6	1.02	0.311
		Average visitation rate	0.19	1	0.661			
		Average number of open flowers	12.99	1	**<0.001**			
		Treatment x Plant age	11.02	3	**0.012**			
Including only treatment and plant age as predictors	10.92	Treatment	0.06	1	0.811	3	-3.31	**0.002**
		Plant age	6.46	3	0.091	4	-0.42	0.676
		Treatment x Plant age	18.01	3	**<0.001**	5	1.82	0.073
						6	1.90	0.061
